# The Uniform of the British Nurse

**Published:** 1917-07-14

**Authors:** 


### THE UNIFORM OF THE BRITISH NURSE.

Of late the question of nurses' uniforms, including collars, cuffs, and belts, has again arisen. The discussion originated, we understand, with the V.A.D.s, with the majority of whom the question of uniform seems to be uppermost. It has been settled, however, at a V.A.D. Committee that they are to continue to wear the regulation collars, cuffs, and belts until it is found that starch is not procurable for laundry purposes. V.A.D.s are allowed to wear a polo collar with the blouse, with or without ties. One of the most experienced of British matrons writes: "I cannot think that trained nurses have any question to ask concerning their uniform. It would be well if none of them condescended to wear variation; this can only be called fancy dress, such as open necks, soft low collars, etc." Another nursing authority declares: "Of course we will make any change in our uniform for national emergencies—should the need arise—but one has first to be told that *substitutes* for starch are not available. Regarding the so-called discomforts of the white collar, no well-trained and disciplined nurse has seriously suggested its change in England for the whims of fashion. With regard to the cuffs, they are not worn when working and cause no inconvenience when off duty. But they do make that professional neatness and finish which go so far in helping to maintain the professional appearance and position which create the desired confidence from the patient. After all, what is uniform for but protection and moral support?"

---

A matron of over thirty years' experience writes: "We hear a good deal in these days of whether the straight linen collar, which English nurses almost invariably wear as part of their uniform, should be abolished for something more comfortable to wear but less pleasing to look at. But for the appearance, the turn-down collar would have superseded the present pattern long ago. I think stiff cuffs are not suitable for nurses when doing things for their patients. Anyone with nursing instincts shudders to see a nurse with large stiff cuffs and huge cuff-links raising a patient in bed, and causing much unnecessary discomfort by digging the cuff-links into the patient's back. We abolished cuffs long ago except at meal-times, and substituted short sleeves made of cambric or nainsook. It seemed more hygienic for nurses to wear the ordinary sleeves of their uniform and the clean linen cuffs which correspond with the collar when taking their own food. We have always preferred the broad linen band of the apron, which keeps it severely in place, to any sort of belt, which appears to us to have a somewhat hard effect. There can be no doubt that a neat and trim appearance is very desirable in a nurse, both from her own and from the patient's point of view."

---

On this point, the *Manchester Guardian*, which recently in a leading article raised the whole question of the nurse's uniform, after affirming that white collars, cuffs, and waist-belts stand for authority, and, as the Lord Chancellor without his wig would be a man of straw, so the nurse in limp collar and no cuffs would lack power, proceeds: "Florence Nightingale affirmed almost passionately the beneficial effects of certain sights and scents on the physical condition of the sufferer; and if these snowy whitenesses, acting first on the body through the nerve of sight, the body in turn acting on the mind—she insisted on this order—can contribute ever so little to recovery, then the case for preserving them is made out."

On the other hand, it is contended that the high bib to the apron, which is very becoming to most people and generally liked, complicates the collar question, for a turn-down collar of any material does not correspond well with this. A looser sort of uniform would be more comfortable to work in, but the more compact uniform, which nurses have adopted, is very convenient for the performance of a nurse's duties, and has so much the advantage as far as appearances are concerned, that it is difficult to believe that nurses will willingly change it. The white sleeves are popular not only because they can be conveniently removed when bare arms are desirable in caring for patients, but because the lower part of the sleeve, being white, looks more artistic than cuffs against the white apron. The general opinion amongst those best entitled to give one is that a nurse's uniform should be kept as becoming as may be, with a fundamental advantage of making its shape and its neatness suitable for the work. No one amongst British nurses objects to the appearance of the present collar, and many have a strong conviction that a soft turn-down collar would not be nearly as becoming to many faces.

Another view maintains that the present collar could certainly be improved upon, but what to suggest in its place few, if any, have made up their minds. The white belt, the same as the apron, is generally popular, and in some hospitals white cuffs are never worn in the wards; the staff on duty always nurse with arms bare up to the elbow, with white linen sleeves to the shoulder. A matron who is highly practical, and whose hospital does her infinite credit, wonders if an overall would be a good thing for nurses to wear—a linen overall with a turn-over collar—wearing a perfectly plain cotton dress underneath, with a low collar. She is of opinion that this, well made, would make a very smart and imposing uniform for nurses, which would then do away with aprons, cuffs, collars, sleeves, and belts. We should welcome opinions on this suggestion, which, on the face of it, seems to be practical. Whether it would be popular is another question.

"A medical officer in charge of a large military hospital of 200 beds" declares his nurses complain of the very high collars they are bound to wear, which play havoc with the skin on their necks. He calls for some nursing authority to come forward and end this ridiculous fashion and allow the nurses to wear something comfortable. We have given the views of various authorities in order to voice nursing opinion comprehensively. A competent critic complains that any straw will be gathered to cause a demoralising effect upon any form of discipline in the nursing world. This critic declares that no other profession would allow its uniform to be trifled with as does ours. We have dwelt on the point of nurses' uniform at some length because there is reason to fear that certain nurses are getting slack, although they cannot be blamed for this question about collars, cuffs, and belts. All fully-trained nurses are rightly satisfied with the compact uniform British nurses generally wear, which is convenient for the performance of a nurse's varied duties, and has much the advantage as far as appearances are concerned. We invite and shall welcome correspondence, experience, and views. The United States nurses wear soft, turn-down collars and cuffs, the latter being taken off and the sleeves turned up when nursing in the wards.

